# MeTEor: an R Shiny app for exploring longitudinal metabolomics data

**DOI:** 10.1093/bioadv/vbae178

**Published:** 2024-11-14

**Authors:** Gordon Grabert, Daniel Dehncke, Tushar More, Markus List, Anke R M Kraft, Markus Cornberg, Karsten Hiller, Tim Kacprowski

**Affiliations:** Division Data Science in Biomedicine, Peter L. Reichertz Institute for Medical Informatics of Technische Universität Braunschweig and Hannover Medical School, Braunschweig, Lower Saxony 38106, Germany; Braunschweig Integrated Centre of Systems Biology (BRICS), Technische Universität Braunschweig, Braunschweig, Lower Saxony 38106, Germany; Division Data Science in Biomedicine, Peter L. Reichertz Institute for Medical Informatics of Technische Universität Braunschweig and Hannover Medical School, Braunschweig, Lower Saxony 38106, Germany; Braunschweig Integrated Centre of Systems Biology (BRICS), Technische Universität Braunschweig, Braunschweig, Lower Saxony 38106, Germany; Braunschweig Integrated Centre of Systems Biology (BRICS), Technische Universität Braunschweig, Braunschweig, Lower Saxony 38106, Germany; Department of Bioinformatics and Biochemistry, BRICS, Technische Universität Braunschweig, Braunschweig, Lower Saxony 38106, Germany; Data Science in Systems Biology, School of Life Sciences, Technical University of Munich, Munich, Bavaria 85354, Germany; Centre for Individualised Infection Medicine (CiiM), Joint Venture Between Helmholtz Centre for Infection Research (HZI) and Hannover Medical School (MHH), Hannover, Lower Saxony 30625, Germany; Department of Gastroenterology, Hepatology, Infectious Diseases and Endocrinology, Hannover Medical School (MHH), Hannover, Lower Saxony 30625, Germany; German Centre for Infection Research (DZIF), Partner Site Hannover-Braunschweig, Hannover, Lower Saxony 38124, Germany; Centre for Individualised Infection Medicine (CiiM), Joint Venture Between Helmholtz Centre for Infection Research (HZI) and Hannover Medical School (MHH), Hannover, Lower Saxony 30625, Germany; Department of Gastroenterology, Hepatology, Infectious Diseases and Endocrinology, Hannover Medical School (MHH), Hannover, Lower Saxony 30625, Germany; German Centre for Infection Research (DZIF), Partner Site Hannover-Braunschweig, Hannover, Lower Saxony 38124, Germany; Braunschweig Integrated Centre of Systems Biology (BRICS), Technische Universität Braunschweig, Braunschweig, Lower Saxony 38106, Germany; Department of Bioinformatics and Biochemistry, BRICS, Technische Universität Braunschweig, Braunschweig, Lower Saxony 38106, Germany; Division Data Science in Biomedicine, Peter L. Reichertz Institute for Medical Informatics of Technische Universität Braunschweig and Hannover Medical School, Braunschweig, Lower Saxony 38106, Germany; Braunschweig Integrated Centre of Systems Biology (BRICS), Technische Universität Braunschweig, Braunschweig, Lower Saxony 38106, Germany

## Abstract

**Motivation:**

The availability of longitudinal omics data is increasing in metabolomics research. Viewing metabolomics data over time provides detailed insight into biological processes and fosters understanding of how systems react over time. However, the analysis of longitudinal metabolomics data poses various challenges, both in terms of statistical evaluation and visualization.

**Results:**

To make explorative analysis of longitudinal data readily available to researchers without formal background in computer science and programming, we present MEtabolite Trajectory ExplORer (MeTEor). MeTEor is an R Shiny app providing a comprehensive set of statistical analysis methods. To demonstrate the capabilities of MeTEor, we replicated the analysis of metabolomics data from a previously published study on COVID-19 patients.

**Availability and implementation:**

MeTEor is available as an R package and as a Docker image. Source code and instructions for setting up the app can be found on GitHub (https://github.com/scibiome/meteor). The Docker image is available at Docker Hub (https://hub.docker.com/r/gordomics/meteor). MeTEor has been tested on Microsoft Windows, Unix/Linux, and macOS.

## 1 Introduction

Advances in high-throughput techniques have revolutionized the generation of molecular biology data. These developments facilitate longitudinal studies to investigate different levels of biological systems and disease-related system changes over time, for example in metabolism and gene regulation (Sperisen *et al.* 2015, Zheng *et al.* 2020, Velten and Stegle 2023). Recent longitudinal metabolomics studies have been able to identify prognostic markers for predicting the severity of diseases such as COVID-19 and hepatitis C virus infection (Sindelar *et al.* 2021, Ali *et al.* 2023). Despite the opportunities offered by longitudinal metabolomics studies, their analysis presents scientists with particular challenges (Velten and Stegle 2023). These encompass a high-dimensional feature space, temporal and intra-individual variations, the occurrence of sample dropouts, and variability in the dependency structure of metabolites across different time points (Metwally *et al.* 2022). In response to these challenges and the growing need for a more profound understanding of longitudinal omics data, a variety of advanced analysis approaches have emerged in recent years (Domanskyi *et al.* 2020, Anžel *et al.* 2022, Bodein *et al.* 2022, Metwally *et al.* 2022, Mor *et al.* 2022, Velten *et al.* 2022, Vasaikar *et al.* 2023). However, incorporating advanced methods into analysis workflows can be a challenge for researchers in fields such as biology, as academic training often does not place much emphasis on programming and statistics (Weissgerber *et al.* 2016, Attwood *et al.* 2019). As a result, researchers may encounter significant obstacles when trying to utilize these tools. To address these challenges and enhance method accessibility, the development and utilization of Shiny applications for analyzing biomedical datasets presents a practical and effective option. Shiny (Chang *et al.* 2024) is a framework that enables the creation of interactive web applications based on R or Python code that can facilitate the analysis and visualization of biological data (Jia *et al.* 2022). In an effort to enhance the exploration of longitudinal metabolomics for researchers in the biomedical field, we introduce MEtabolite Trajectory ExplORer (MeTEor). MeTEor is an open-source R (R Core Team 2023) Shiny App designed to provide a user-friendly and efficient research tool for longitudinal data exploration in metabolomics. MeTEor includes methods for dimensionality reduction, machine learning, statistical testing, network analysis, and pathway enrichment. Parts of the data manipulation and visualizations in MeTEor were done using *tidyverse* packages (Wickham *et al.* 2019). MeTEor also incorporates TCAM (Mor *et al.* 2022), a tensor factorization method developed to uncover insights beyond individual variations in longitudinal data. It allows for efficient dimensionality reduction while preserving the inherent geometric and statistical characteristics of the data. The results from TCAM can be contrasted with those obtained from conventional principal components analysis (Lê *et al.* 2008, Kassambara and Mundt 2020). To refine the identification of predictive or discriminatory metabolites, a combination of prediction and statistical testing methods is integrated, including random forests (Liaw and Wiener 2002, Kuhn 2008, Robin *et al.* 2011), logistic regression, XGBoost (Chen *et al.* 2024), linear mixed models (Bates *et al.* 2015), repeated measures, and mixed ANOVA (Kassambara 2023a,b), along with tests for statistical assumptions (Lüdecke *et al.* 2021). After assessing feature importances, the most important metabolites can be explored in depth using data visualization tools. MeTEor offers a range of visualization techniques, including ridge plots (Wilke 2024), heatmaps (Galili *et al.* 2018, Kolde 2019), volcano plots (Ritchie *et al.* 2015, Blighe *et al.* 2024), and networks (Almende *et al.* 2019, Haslbeck and Waldorp 2020, Kuhn *et al.* 2022, Csárdi *et al.* 2024). The MetaboAnalyst ID Conversion (Pang *et al.* 2021) can be used to query the IDs of interesting metabolites, followed by the execution of pathway analysis using KEGGREST (Tenenbaum and Maintainer 2023) and generation of modified URLs to interactive KEGG pathways (Kanehisa *et al.* 2023).

## 2 Use case

**Figure 1.**
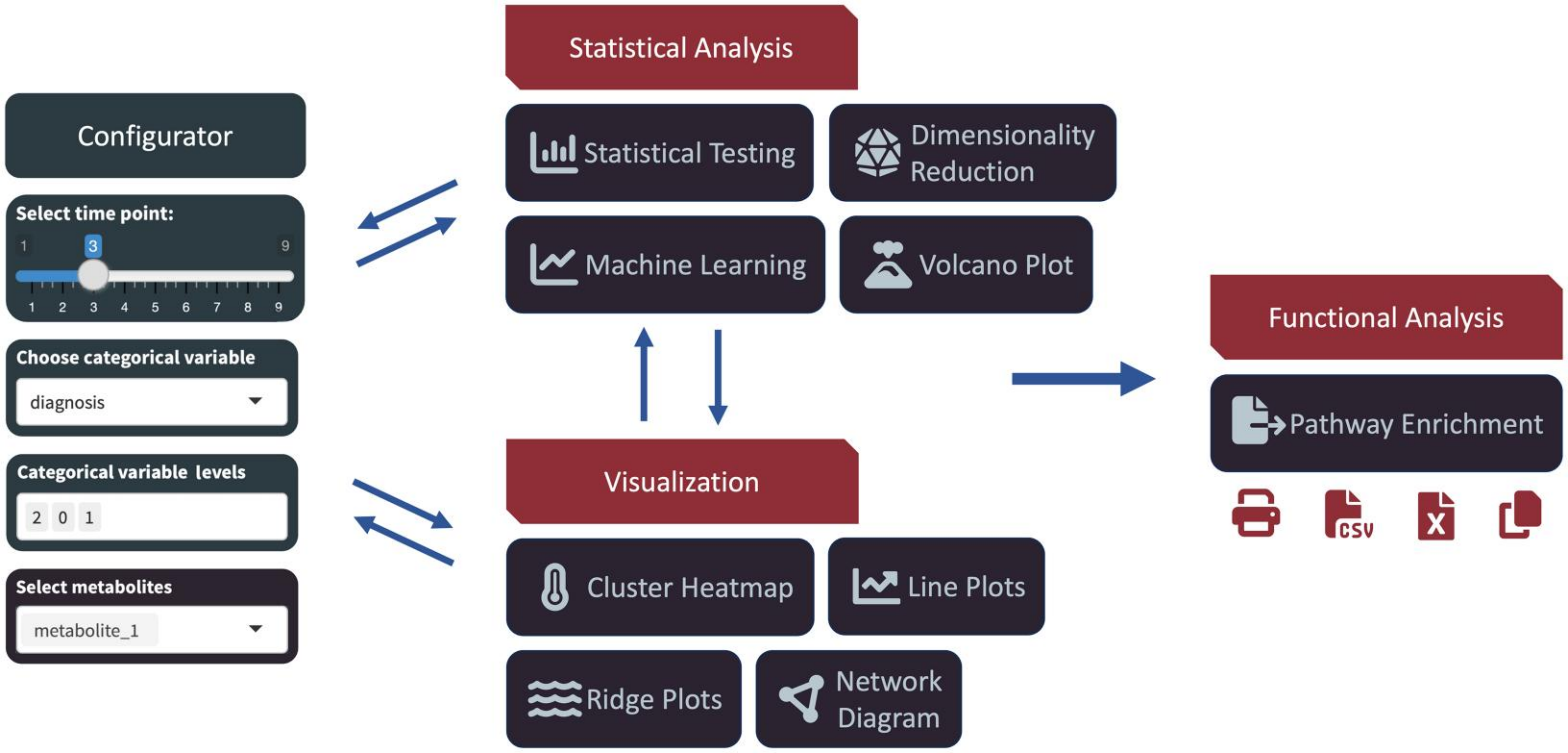
Overview of the various techniques offered by MeTEor: on the left side is the Configurator, enabling a targeted analysis. It is possible to choose a specific time point, select a categorical variable, the levels of the categorical variable, and pick multiple metabolites. The selection is shared across the different methods, facilitating a seamless exploratory analysis. In our example analysis, the loaded data undergoes statistical analysis to identify interesting metabolites that exhibit changes between time points or categories. The chosen metabolites are then subjected to further analysis using diverse visualization techniques such as line plots or heatmaps. Lastly, an enrichment analysis is conducted, revealing pathways with a high prevalence of the selected metabolites, which can then be exported to multiple data formats.

MeTEor provides a comprehensive set of visualization and analysis tools, shown in Figure 1. To showcase its capabilities, we replicated the analysis conducted in the study “Plasma Metabolome Alterations Discriminate between COVID-19 and Non-COVID-19 Pneumonia” (More *et al.* 2022). The original study involved comparing venous and arterial blood samples of patients. Before being loaded into MeTEor, the data needs to be converted into the standard MeTEor long format, containing ID, timepoint (in this case venous and arterial), metabolites, their values, and one or more categories. Additional details regarding the data preparation process can be found in the supplementary materials accompanying the software documentation. Using the MeTEor Configurator, we selected from the “diagnosis category” the condition *COVID-19 infected*. We performed a repeated measures ANOVA to pinpoint metabolites exhibiting significant differences between venous and arterial blood samples. We successfully identified four of the six most significant metabolites highlighted in the prior study: *RI1028.76*, *RI2473.8*, *RI3150.76*, and *RI1021.65*. Discrepancies can be attributed to the more tailored nature of their model, accommodating nested effects, in contrast to our more general approach. In addition, leveraging the implemented XGBoost classifier, we predicted COVID-19 outcomes (*recovered* versus *deceased*) and identified crucial metabolites for classification. The model attains an AUC of 78.3%, demonstrating a predictive capacity for disease severity comparable to the top-performing models identified by More *et al.* (2022). The difference in classification accuracy is most likely due to usage of Synthetic Minority Oversampling Technique (SMOTE) (Chawla *et al.* 2002) by More *et al.* to address imbalances in *recovered* versus *deceased* COVID-19 cases. MeTEor transparently presents key predictors, allowing for detailed scrutiny of significant metabolites. Notably, three of the four pivotal features from the original study—*RI1150.81*, *RI1532.53*, and *RI1557.73*—are among the critical features we identified. Using the MetaboAnalyst ID Conversion (Pang *et al.* 2021) to retrieve the IDs for these metabolites, MeTEor identifies the pathways where the metabolites are most prevalent. MeTEor generates modified URLs to interactive KEGG pathways that highlight these identified metabolites, providing a clear visual representation of their roles and interactions. This feature significantly enhances the understanding of their biological significance and functional context.

## 3 Limitations

Currently, the absence of implemented batch correction methods represents a limitation in data preprocessing. In addition, manual user input is required to obtain identifiers for relevant metabolites from the MetaboAnalyst service (Pang *et al.* 2021), introducing a slight delay in the analysis workflow. As demonstrated in the results section, MeTEor enables rapid general analysis, and the option to export results in various formats enhances the ability to conduct customized follow-up analyses.

## 4 Conclusion

MeTEor is a user-friendly tool that provides a comprehensive overview of longitudinal metabolomics data. Our application of MeTEor to real-world data demonstrates is ability to reproduce results from previously published research. Moving forward, the future goals for MeTEor include the integration of additional functionalities for enrichment analyses and the seamless incorporation of MeTEor into existing metabolomics workflows.

## Data Availability

The data used for the MeTEor demonstration is available directly in the R package and in the Shiny app.
